# Improving reproducibility and reusability in the Journal of Cheminformatics

**DOI:** 10.1186/s13321-023-00730-y

**Published:** 2023-06-30

**Authors:** Charles Tapley Hoyt, Barbara Zdrazil, Rajarshi Guha, Nina Jeliazkova, Karina Martinez-Mayorga, Eva Nittinger

**Affiliations:** 1 Laboratory of Systems Pharmacology, Harvard Medical School, Boston, USA; 2 EMBL-EBI, Hinxton, England; 3 Vertex Pharmaceuticals, Boston, USA; 4 Ideaconsult Ltd, Wigan, UK; 5 National Autonomous University of Mexico, Mexico City, Mexico; 6 Medicinal Chemistry, Research and Early Development, Respiratory and Immunology (R&I), BioPharmaceuticals R&D, AstraZeneca, Gothenburg, Sweden

Reproducibility is essential to independently determine the veracity and integrity of scholarly work. Researchers in the life and natural sciences continue to struggle to achieve reproducibility due to many factors and facets of modern research including methodologies [1], data sharing [2], problematic incentive structures [3], widespread systematic issues with peer review [4–6], and others. Together, they culminate in what is colloquially known as the "reproducibility crisis" [7]. Further, researchers in the computational life and natural sciences struggle to achieve reusability with the workflows, algorithms, and code that they produce [8].

As an initial step towards promoting and improving reproducibility and reusability, the *Journal of Cheminformatics* has adopted relatively progressive policies for code sharing (https://jcheminf.biomedcentral.com/submission-guidelines/preparing-your-manuscript/software). However, many challenges remain in promoting code reproducibility, reusability, and ultimately, utility. Some concrete examples of these challenges include code that is:

1. **Only partially made available.** Most computational workflows include data download, processing, analysis, summarization, and visualization which can be implemented as a combination of programming languages, frameworks, and web applications. Without each step being made explicit with either code or other structured workflow definitions (e.g., for KNIME), neither a full assessment, reproduction, nor reuse can be attempted.
2. **Not licensed.** Unlicensed code can neither be modified nor redistributed. Ideally, an academic work uses an Open Source Initiative (OSI)-approved license to promote reuse, improvement, and redistribution.
3. **Not possible to install automatically**. Installation should be standardized, system-agnostic, simple, and automated. Ideally, code should be packaged so it can be installed automatically using the infrastructure available for each programming language (e.g., using *pip* for Python code). A related issue is that code residing in Jupyter notebooks can often neither be easily installed nor reused. Ideally, code should be packaged then called from Jupyter notebooks to illustrate high-level workflows, not to implement the workflows in Jupyter notebooks themselves.
4. **Not (well) documented**. Code should be documented in order to help average users install and run the code that produced the results of a manuscript. Exceptional work documents how to extend the code and apply it to new data sets and scenarios further than what is described in its manuscript. Documentation should use language-specific documentation tools (e.g., Roxygen for R, Javadocs for Java, Sphinx for Python). Further, it should either be built and hosted (e.g., on ReadTheDocs or GitHub Pages) or include simple, reproducible build instructions.
5. **Non-conformant to community standard style guidelines**. Code itself constitutes the methods section of a research article. Therefore, it should be written with the expectation that it is to be read. Each programming language has its own community style standards, such as PEP-8 for Python. Linting tools like *black* (for Python) are an easy stop-gap for automatically formatting code in a clean, consistent way. Further, conformance to a given linter's standard can be automatically checked and potentially even prescribed by the journal.
6. **Not externally permanently archived using an appropriate repository like Zenodo or FigShare**. While the *Journal of Cheminformatics* has already taken the initiative to archive repositories in https://github.com/jcheminform by forking (if the author's repository originated from GitHub) or mirroring (if the repository originated from another version control system like GitLab), it's possible that GitHub could lose popularity similarly to preceding tools like SourceForge. The likelihood that Zenodo or FigShare disappears is much less, considering their stated missions are related to providing permanent archival of scientific artifacts. Therefore, code should be archived by the authors using such archival systems. For example, GitHub has a plugin (https://docs.github.com/en/repositories/archiving-a-github-repository/referencing-and-citing-content) to automate archiving to Zenodo on creation of "releases".

Many researchers in the computational sciences have not trained in software engineering as part of their schooling nor ongoing professional development. Given that each of these challenges contain many facets, it would be daunting for most researchers to address them all at once. Therefore, their essences have been distilled into the following seven questions that can be asked and easily reasoned about a given code repository:

1. Does the repository contain a LICENSE file in its root?
2. Does the repository contain a README file in its root?
3. Does the repository contain an associated public issue tracker?
4. Has the repository been externally archived on Zenodo, FigShare, or an equivalent that is referenced in the README?
5. Does the README contain installation documentation?
6. Is the code in the repository installable in a straight-forward manner?
7. Does the code in the repository conform to an external linter (e.g., *black* for Python)?

While these questions by no means constitute a complete guide towards addressing the aforementioned challenges, they are a gentle first step towards normalizing code review as part of typical peer review that focus on simple improvements that are often overlooked.

We are starting a pilot to include an evaluation of these seven questions by an additional reviewer in the *Journal of Cheminformatics*. This pilot will both investigate the status of code repositories corresponding to research articles upon initial submission to the journal as well as to evaluate how perceptive authors are to making improvements based on the feedback generated from applying the questionnaire. In parallel, we plan to develop improved guidelines for authors to address the issues covered by the questionnaire proactively as well as for code reviewers to apply them. Finally, we hope this pilot encourages the community to improve its standards over time and to better educate researchers and reviewers.

## Data Availability

Not applicable.
